# Healthcare access difficulty due to fear of COVID-19 infection among women of reproductive age: multilevel mixed-effects logistic regression analysis

**DOI:** 10.4314/ahs.v26i1.3

**Published:** 2026-03

**Authors:** Charles Natuhamya

**Affiliations:** Makerere University School of Public Health, P.O. Box 7062, Kampala-Uganda

**Keywords:** COVID-19, Fear of COVID-19, Healthcare access difficulty, Kenya, Women of reproductive age

## Abstract

**Introduction:**

The coronavirus disease of 2019 (COVID-19) pandemic left lasting effects in all sectors globally. Particularly among women of reproductive age, it has instilled difficulty of accessing healthcare, which has been linked to fear of being exposed to the virus at health facilities. However, most studies especially in East Africa have narrowed COVID-19 related research to qualitative reviews and descriptive analyses due to inadequacy of COVID-19 data. The purpose of this study was to determine the relationship between health facility visiting and healthcare access difficulty due to fear of COVID-19 at health facilities, while exploring both individual and contextual determinants.

**Methods:**

The current study applied a multilevel logistic mixed-effects regression model to account for both individual and contextual dimensions in the study.

**Results:**

Overall, 30.0% of the women reported difficulty in accessing healthcare. Women who had visited a health facility in the past 4 weeks of the survey and had health insurance were associated with less healthcare access difficulty; (AOR= 0.73; 95% CI, 0.57-0.95) and (AOR= 0.74; 95% CI, 0.59-0.94) respectively. On the contrary, women who watched television, in households that had partially and completely lost household income due to the virus were associated with more difficulty; (AOR= 1.38; 95% CI, 1.10-1.74), (AOR= 2.09, 95% CI, 1.51-2.88) and (AOR= 2.40, 95% CI, 1.65-3.47) respectively. About 72% of the total variance in healthcare access difficulty was attributable to differences in enumeration areas (ICC= 0.72; 95% CI, 0.66-0.76), with less significant individual level contribution.

**Conclusion:**

Interventions that influence routine health facility visits can boost healthcare access among women. Enumeration area-specific interventions may be more effective.

## Introduction

Coronavirus disease of 2019 (COVID-19) has directly and/or indirectly threatened many people globally^1^, leaving a significant psychological impact on the lives of many^2^. African countries have witnessed less queues of patients with acute respiratory symptoms (low healthcare seeking), which may explain the low rates of COVID-19 symptomatic cases and infection^3^. Although Kenya (the current study area) implemented nationwide curfew from 7 pm to 5 am and movement restrictions^4^,^5^, access to healthcare was majorly affected by COVID-19 in several other ways besides these control measures^6^.

A significant number of people reported difficulty in accessing health services^7^ of which fear of COVID-19 infection was the dominant barrier to this^6^, ^8^-^10^. Access to healthcare for women has been a critical dimension impaired by the COVID-19 pandemic^11^. For instance, a study in Kenya reported that about 40% of women of reproductive age were hesitant to visit a health facility due to fear of being exposed to COVID-19 at health facilities^12^. Unfortunately, the fear reduced the public's ability to fight the virus as it created panic and social crises that pose as challenges to the medical recommendations for COIVID-19 prevention and control^13^. Ethologically, fear leads to avoidance behavior^14^ like avoidance of healthcare seeking in the case of this study. Reduced healthcare seeking may lead to poor health outcomes for other diseases besides COVID-19 due to reduced access to health facilities^15^.

A major limitation in COVID-19 research especially in low and middle income countries has been inadequacy of data^4^, ^16^, ^17^ limiting research publications to qualitative reviews and descriptive analyses. Hence, this study in particular will add area or contextual analysis to the existing research in the focus area. This study particularly aimed at determining the relationship between health facility visiting and healthcare access difficulty due to fear of COVID-19 infection at health facilities, while exploring both individual and contextual determinants in a multilevel study design setting.

## Methods

### Data source and sampling design

Version 2.0 of the Performance Monitoring for Action (PMA) Kenya Phase 2 household and female survey dataset was used in this study. Performance Monitoring for Action (PMA) is a latter version of PMA2020 surveys in Kenya which collects routine data on key global indicators in family planning and reproductive health. The first round of data collection targeted a sample size of 120 EAs that were selected from the master sample frame of the Kenya National Bureau of Statistics (KNBS), a representative at both the national and county levels for urban and rural areas. The KNBS selected 9 counties (Nairobi, Kilifi, Nandi, Nyamira, Kiambu, Bungoma, Siaya, Kericho and Kitui) from 47 counties at the first stage, using probability proportional to size. Thirteen EAs were sampled in each of the 6 counties and 14 EAs from each of the remaining 3 counties (Kiambu, Bungoma and Nairobi). EAs created during the 2009 Kenya Population and Housing Census were the primary sampling units for the survey. The EAs were selected systematically with probability proportional to size with urban/rural stratification in the 9 counties. The KNBS provided the selection probabilities for the PMA2020 sampled clusters that facilitated constructing weights^18^.

Cross-sectional and panel Household and Female surveys are conducted annually in Kenya, with panel follow-up in years 2 and 3. Phase 2 of PMA survey was based on a multi-stage (two-stage) cluster design, with stratification at the urban and rural level or sub region. Within each urban/rural or sub regional stratum, EAs (primary sampling units) were selected using probability proportional to size (PPS) method. All households were listed and mapped per EA prior to baseline data collection. PMA applied an open panel design which enrolled new eligible women at annual follow-ups. Households selected at baseline and still residing in the EA were followed up in subsequent rounds. Adolescents in selected households aged 14 years in the previous round were enrolled in the panel as 15-year-olds. Women who were previously aged 49 years were not interviewed in the subsequent round. Households that moved out of the EA since baseline were considered lost-to-follow-up^19^.

This study used data on 5,005 women of reproductive age who attempted to access health services within 4 months prior to the survey. To access the dataset, a PMA account was registered on the PMA website detailing a brief description of the study. Upon approval, the dataset was made available for download and use for free.

### Study variables

**Figure 1:**
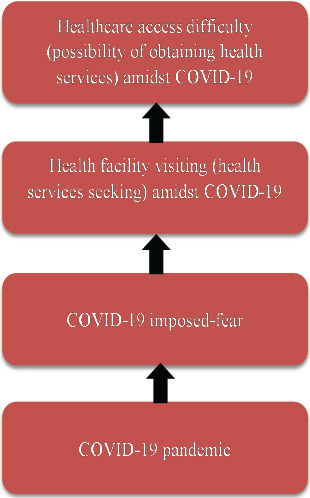
A Link between COVID-19 Pandemic and Healthcare Access Difficulty

The outcome variable was healthcare access difficulty due to fear of COVID-19 infection at health facilities, a binary categorical variable (had difficulty or not). The exposure variables in this study included; health facility visiting, possession of health insurance, COVID-19 concern, watching television, COVID-19 household income loss, type of place of residence, among others. In this study, access to healthcare was conceptualized as potential access, defined as the process of accessing care as opposed to realized access, that is, the actual use of healthcare services. Details are shown in Figure 1.

### Statistical analysis

Before any analysis was conducted, data were explored, some variables regrouped to account for variation in the categories while other variables that were not of interest to the research problem were eliminated. Categorical variables of women, households and EAs were presented as frequencies and proportions by healthcare access difficulty using Pearson Chi-square test. Collinearity was assessed among independent variables using a correlation matrix at a cut-point of correlation coefficient of 0.4. All analyses were conducted using Stata 15.0 (StataCorp, College Station, TX). The level of statistical significance was 5% for all statistical tests. Level-specific weights were applied at each hierarchy during analysis. Analyses were conducted by complete case analysis since the level of missing data was below 10%^20^, a level that is nearly devoid of biased results.

Bivariate level of analysis was conducted using bivariate logistic mixed-effects regression models. Only variables that were associated with healthcare access difficulty at p-value of 0.20 were considered for multi-variable modelling. Backward step elimination criteria was applied to exclude predictors that were not significant at multi-variable level, one at a time and only variables that were significant were considered as main predictors in the multi-variable model.

Four multilevel logistic mixed-effects regression models were applied to the data. Model 1 (null model) included the random intercept to assess cluster (EA) contribution to the variation in healthcare access difficulty before including fixed effects. Model 2 adjusted for individual level variables in addition to the random intercept. Model 3 adjusted for household level variables in addition to variables in model 2. Finally, model 4 included the EA-level variable in addition to variables adjusted for in model 3. While reporting, fixed effects were presented as adjusted odds ratio (AOR) while random effects were reported by variance components: variance and intraclass correlation coefficient (ICC). Individual and household level variables were included as fixed effects while the enumeration area variable was added as the random effect.

### Multilevel mixed-effects models

Multilevel mixed-effects logistic regression models were specified to account for contextual within-enumeration area correlations. The model is represented as below:


$$\ln\left[\frac{p_{ijk}}{1-p_{ijk}}\right]=\beta_{0}+\beta_{1}X_{ijk}+\eta_{k}+\xi_{jk}$$

*ln* is the natural logarithm.
*p_ijk_* is probability of occurrence of the outcome (healthcare access difficulty) for *i^th^* woman at household *j* in EA *k*.
*β*_0_ is the mean log-odds of healthcare access difficulty across household and EA.
*X_ijk_* is a level 1 covariate for the *i^th^* woman in household *j* and EA *k*.
*β*_1_ represents the slope associated with *X_ijk_* which represents the relationship between the level-1 (individual woman) covariates and the log-odds of healthcare access difficulty.
*η_k_* is the random effect for EA *k*.
*ξ_jk_* is the household random effect.

It should be noted that the random effects are independent and identical to each other and are assumed to be normally distributed with mean of zero and variances *σ_η_* and *σ_ξ_*.

### Cluster variation

The extent to which individuals within the same cluster (EA) are more similar to each other than they are to individuals in different EAs was measured by intraclass correlation coefficient (ICC)^21^-^23^. A higher proportion of the ICC was linked to a higher general contextual effect^24^. The formula for the ICC is presented as below:


$$ICC=\frac{V_{A}}{V_{A}+\frac{\pi^{2}}{3}}$$

where *V*_*A*_ is the cluster (area) level variance and $\frac{\pi^{2}}{3}$ is a scalar that corresponds to the individual level variance.

## Results

### Regional Difficulty in Healthcare Access

**Figure 2:**
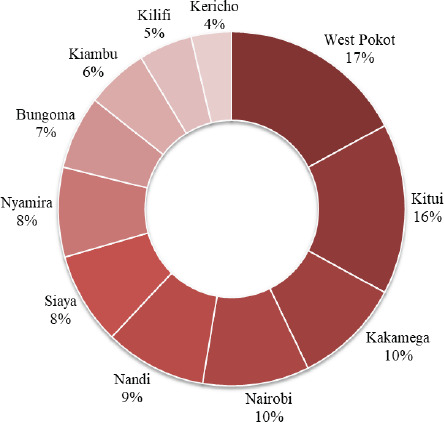
Proportion of Access to Healthcare Difficulty by Geographical Region

The national proportion of women who experienced difficulty in accessing healthcare was 30.0% on average. West Pokot and Kericho regions had the highest and lowest proportions (17.2% and 3.8%) respectively as shown in Figure 2.

The Chi-square test results indicate that a significantly higher proportion of women did not visit a health facility within the past 4 weeks of the survey (39.1%), did not have health insurance (31.2%), and were not concerned about COVID-19 (43.6%). Details of these results are in Table 1.

**Table 1:** Background characteristics of women aged 14 to 49 years by healthcare access difficulty

Variables	n (%)	Categories	Healthcare access difficulty, n (%)		p
			No	Yes	
Visited health facility	1,201 (24.0)	No	731 (60.9)	470 (39.1)	<0.001
	3,804 (76.0)	Yes	2776 (73.0)	1028 (27.0)	
Has health insurance	3450 (68.9)	No	2375 (68.8)	1075 (31.2)	0.005
	1555 (31.1)	Yes	1132 (72.8)	423 (27.2)	
COVID-19 concern	149 (3.0)	Not concerned	84 (56.4)	65 (43.6)	<0.001
	152 (3.0)	A little concerned	131 (86.2)	21 (13.8)	
	1075 (21.5)	Concerned	812 (75.5)	263 (24.5)	
	3629 (72.5)	Very concerned	2480 (68.3)	1149 (31.7)	
Watched television	2303 (46.0)	No	1689 (73.3)	614 (26.7)	<0.001
	2702 (54.0)	Yes	1818 (67.3)	884 (32.7)	
COVID-19 householdincomeloss	1032 (20.6)	None	790 (76.6)	242 (23.4)	<0.001
	1252 (25.0)	Partial	1856 (68.2)	865 (31.8)	
	2721 (54.4)	Complete	861 (68.8)	391 (31.2)	
Type of place of residence	1477 (31.5)	Urban	1008 (68.3)	469 (31.7)	0.150
	3215 (68.5)	Rural	2261 (70.3)	954 (29.7)	

p-values were obtained by Chi-square test

### Factors associated with difficulty of accessing healthcare due to fear of COVID-19 infection

The random effect of the null model (model 1) showed a statistically significant variation in healthcare access difficulty across EAs (variance= 8.17; 95%CI, 6.31-10.57). The ICC value for healthcare access difficulty between EAs (ICC= 0.72; 95%CI, 0.66-0.76) indicates that 72% of the total variance in healthcare access difficulty was attributable to differences in EAs. However, the ICC did not change significantly across the 4 models. This indicates that fixed effects did not considerably contribute to the variation in healthcare access difficulty. Model 4 was selected as the final model since it had the lowest AIC value (3414.42).

The fixed effects of the final model indicate that women who had visited a health facility in the past 4 weeks of the survey (AOR= 0.73; 95% CI, 0.57-0.95) and had health insurance (AOR= 0.74; 95% CI, 0.59-0.94) were significantly associated with reduced difficulty of access to healthcare. On the contrary, women who had watched television (AOR= 1.38; 95% CI, 1.10-1.74) were associated with more difficulty of access to healthcare. Also, households whose income was partially lost (AOR= 2.09, 95% CI, 1.51-2.88) and completely lost (AOR= 2.40, 95% CI, 1.65-3.47) due to COVID-19 effects were more likely to face difficulty in accessing healthcare (Table 2).

**Table 2:** Factors associated healthcare access difficulty due to fear of COVID-19

Variables	Model 1	Model 2	Model 3	Model 4
	AOR (95%CI)	AOR (95%CI)	AOR (95%CI)	AOR (95%CI)
***[Fixed effects]***				
**Visited health facility**				
No (ref)	-	1.00	1.00	1.00
Yes	-	0.78 (0.60-1.00)*	0.75 (0.58-0.97)*	0.73 (0.57-0.95)*
**Has health insurance**				
No (ref)	-	1.00	1.00	1.00
Yes	-	0.73 (0.58-0.91)*	0.74 (0.59-0.93)*	0.74 (0.59-0.94)*
**COVID-19 concern**				
Unconcerned (ref)	-	1.00	1.00	1.00
Concerned	-	2.78 (1.18-6.52)*	2.56 (1.10-5.98)*	2.32 (0.98-5.49)
**Watched television**				
No (ref)	-	1.00	1.00	1.00
Yes	-	1.38 (1.11-1.72)*	1.36 (1.09-1.70)*	1.38 (1.10-1.74)*
**C OVID-19 household income loss**				
None (ref)	-	-	1.00	1.00
Partial	-	-	1.98 (1.45-2.71)**	2.09 (1.51-2.88)**
Complete	-	-	2.37 (1.65-3.39)**	2.40 (1.65-3.47)**
**Type of place of residence**				
Urban (ref)	-	-	-	1.00
Rural	-	-	-	1.13 (0.59-2.18)
***[Random effects]***				
EA variance	8.17 (6.31-10.57)	8.22 (6.35-10.64)	8.28 (6.40-10.72)	8.54 (6.56-11.13)
ICC	0.72 (0.66-0.76)	0.71 (0.66-0.76)	0.71 (0.66-0.77)	0.72 (0.67-0.77)
LR	2464.37	2405.28	2377.57	2223.48
AIC	3648.44	3632.38	3611.96	3414.42

* p<0.05

** p<0.001

AOR: Adjusted odds ratio, CI: Confidence interval, LR: Likelihood ratio test, AIC: Akaike information criterion

## Discussion

In this study, we aimed to determine the relationship between health facility visiting and healthcare access difficulty due to fear of COVID-19 infection at health facilities in the past 4 months of the survey, while exploring both the individual and contextual dimensions. Visiting a health facility and ownership of health insurance were associated with less difficulty in accessing healthcare. COVID-19 related household income loss, COVID-19 concern and watching TV were associated with higher healthcare access difficulty.

The study results indicated a lower likelihood of COVID-19 related healthcare access difficulty among women that had visited a health facility in the past 4 weeks of the survey compared to their counterparts. This could be due to access of credible information from health workers which reduces the fear of COVID-19 infections and increases healthcare seeking^25^, ^26^.

Women who had health insurance were associated with less difficulty of healthcare access due to COVID-19 infection related fear. This finding could imply that women whose health is insured feel safer and more protected against COVID-19 risks outside their homes due to the health insurance coverage^27^ hence less fear of COVID-19 infection.

Media exposure in this study concentrated on women who had watched TV since the main source of information on COVID-19 according to research that was carried out during the COVID-19 pandemic in Kenya was a TV^28^. Findings from this current study agree with earlier reports which indicated that media exposure increased fear^29^ as the media usually put much attention to critical messages portraying failure of global health systems to deal with COVID-19 infections^2^. Such unreliable information is normally provided by the vast uncontrolled media sources especially the social media and TV programs^30^. Hence, to curb widespread misconceptions and anecdotal reports, community leaders and other stakeholders need to sensitize communities especially those in rural settings about COVID-19 and promote reliable information issued by health authorities^31^.

Healthcare access difficulty increased with increase in household income loss. This is in relation to a study which found out that income loss was more common among participants that had reported higher frequencies of feelings of fear during the COVID-19 pandemic^32^. Income loss due to COVID-19 was partly due to reduced daily income, wages and employment^15^.

According to the mixed effects model results, individual and household characteristics (fixed effects) did not significantly change cluster variation in the outcome. The ICC at EA level was considerably high implying a generally higher contextual effect^24^ suggesting that EA-specific intervention would be more effective compared to any other level-specific interventions^33^. Similarly, a study in Uganda showed a greater cluster variation at area level compared to individual level^34^ indicating a greater contribution of the contextual dimension in the outcome.

## Conclusion

Visiting a health facility was associated with less difficulty in accessing healthcare due to fear of COVID-19 at a health facility. Interventions that influence routine health facility visits can boost healthcare access among women. EA-specific interventions may be more effective.

## Data Availability

The datasets analyzed in this study are publicly available for access and download by PMA at: https://www.pma-data.org/data/request-access-datasets
